# Does autophagy play a key role in the protective effect of oleic acid against oxidative stress in endothelial cells?

**DOI:** 10.1007/s11010-025-05410-z

**Published:** 2025-10-15

**Authors:** Ana García-Aguilar, Olga Palomino, Adrián González, Carlos Guillén, María S. Fernández-Alfonso, Luis Goya

**Affiliations:** 1 Department of Pharmacology, Pharmacognosy and Botany, Faculty of Pharmacy, University Complutense of Madrid, Ciudad Universitaria S/N, 28040 Madrid, Spain; 2 Department of Biochemistry and Molecular Biology, Faculty of Pharmacy, University Complutense of Madrid, Ciudad Universitaria S/N, 28040 Madrid, Spain; 3 IdISSC, Madrid, Spain; 4 Department of Metabolism and Nutrition, Institute of Food Science, Technology and Nutrition (ICTAN—CSIC), 28040 Madrid, Spain

**Keywords:** Oleic acid, Autophagy, Oxidative stress, Endothelial cells, Atherosclerosis

## Abstract

Endothelial dysfunction is a primary cause of cardiovascular complications that lead to atherosclerosis, while oxidative stress has been highligthted as one mechanism involved in endothelial dysfunction. Prevention of oxidative stress may then be a strategy to avoid endothelial dysfunction and cardiovascular disease. As the ability of oleic acid of reducing reactive oxygen species and related oxidative stress has been shown, other potential cellular mechanisms that could be responsible for the protective effect have to be evaluated. Autophagy is considered a cellular adaptive response under stressful conditions; thus, its role in the protective mechanism of oleic acid in stressed endothelial (EA.hy926) cells was assessed. To that end, cell viability and markers of oxidative status, such as reactive oxygen species, reduced glutathione, glutathione peroxidase, and reductase were evaluated. Moreover, the expression of several key autophagy-related proteins, such as microtubule-associated protein 1 light chain 3 beta and ubiquitin-binding protein p62/sequestosome 1, were investigated. The results showed that oleic acid within the micromolar range stimulated autophagy. However, when autophagy was inhibited in endothelial cells under oxidative stress, changes in the chemoprotective effect of oleic acid were minimal. These results suggest a limited contribution of autophagy to the protective effect of oleic acid under conditions of severe oxidative stress.

**Supplementary Information:**

The online version contains supplementary material available at 10.1007/s11010-025-05410-z.

## Introduction

One of the initial steps of endothelial dysfunction, which is the primary cause of cardiovascular complications involved in the pathogenesis of atherosclerosis, diabetes, and related metabolic disorders, is the damage to endothelial cells that form the internal coating of blood vessels [1]. Endothelial dysfunction can be provoked by conditions such as oxidative stress, hyperglycemia, hyperlipidemia, and increased proinflammatory cytokines [2, 3]. Oxidative stress, an imbalance of the redox status within the cell that results in a pro-oxidant condition by overproduction of reactive oxygen species (ROS), is one of the most frequent mechanisms of damage to cells and tissues, including the endothelium [4]. Despite the efficient cellular antioxidant systems, such as reduced glutathione (GSH), glutathione peroxidase (GPx), glutathione reductase (GR), glutathione-S-transferase (GST), catalase (CAT), and superoxide dismutase (SOD), a large or long-lasting generation of ROS may saturate these defense mechanisms and cause oxidative stress that induces inflammation and mitochondrial dysfunction, ultimately leading to apoptosis [4, 5]. Thus, preventing oxidative stress is a useful strategy for preventing endothelial dysfunction and apoptosis.

Although free fatty acids (FFAs) are vigorous sources of energy for cells by producing ATP through beta-oxidation, elevated plasma FFAs, such as oleic (18:1 n-9, OA) [6] but especially saturated non-esterified fatty acid palmitic acid (16:0), may provoke vascular endothelial dysfunction [7–10]. Expanded adipose tissue has been suggested to be a strong risk factor for the development of cardiovascular diseases because of its fatty acid secretory capacity, and the combined elevated release of FFAs by lipoprotein lipase and adipokines secreted by adipose tissue in obese individuals might be associated with vascular dysfunction and the development of atherosclerosis [9]. In contrast, lower concentrations of OA are not only necessary for energy storage and production as well as for turnover in the cell membrane lipid bilayer but may also be beneficial for maintaining endothelial function. We recently reported that OA concentrations in the lower micromolar range protect cultured endothelial cells (EA.hy926) against chemically induced oxidative stress by reducing ROS and enhancing antioxidant defenses [11]. In our initial study on the effect of FFAs on endothelial function, we primarily addressed the response of the cellular antioxidant defense system to oxidative stress, but we also inferred potential involvement of additional protective mechanisms, such as mitochondrial oxidative phosphorylation (OxPhos), cyclic AMP/protein kinase A (cAMP/PKA) signaling, and autophagy [11]. In line with this, Guo and co-workers demonstrated that curcumin activates autophagy and attenuates oxidative damage in EA.hy926 cells through the protein kinase B (Akt)/mammalian target of rapamycin (mTOR) pathway [12]. Furthermore, a recent study showed that an extract from Sambucus nigra exerts protective effects on dysplastic oral keratinocytes by triggering autophagy [13]. Moreover, we have previously demonstrated that the aqueous extract of Sambucus nigra fruit promotes neuroprotection in neuronal-like SH-SY5Y cells, mainly due to its antioxidant capacity and, to a lesser extent, through the activation of autophagy [14]. Autophagy is revealed as a crucial, conserved cellular process in eukaryotes to maintain homeostasis by recycling damaged components, and its dysregulation is linked to various diseases, making it an important adaptive stress response for cell survival [15, 16]. The mTOR complex 1 (mTORC1) and AMP-activated protein kinase (AMPK) pathways oppositely regulate autophagy. Firstly, when energy is abundant, mTORC1 is active and inhibits autophagy by phosphorylating Unc-51-like autophagy-activating kinase 1 (ULK1) at multiple sites, including serine 757. Moreover, under energy stress conditions, AMPK is active and promotes autophagy by inhibiting mTORC1 and activating ULK1 by phosphorylating it at its serine 555 position [17].Microtubule-associated protein 1 light chain 3 beta (LC3B) plays a key role in autophagy by facilitating autophagosome development and maturation. LC3B is widely used as a marker for autophagic activity, as the expression of its lipidated form, LC3B-II, is correlated with autophagy induction. Moreover, the ubiquitin-binding protein p62/sequestosome 1 (SQSTM1) protein directly ubiquitinates proteins or damaged organelles to LC3 and GABARAP family proteins, thus representing the connection between the autophagy pathway and the ubiquitin–proteasome system. The p62/SQSTM1 protein is degraded by autophagy itself so that, when autophagy is blocked, through the use of an autophagy inhibitor (chloroquine or bafilomycin A1), p62 protein expression increases, thus representing a useful biomarker to analyze autophagic flux [17, 18].

The aim of this study was to investigate the direct protective effects of oleic acid (OA) on endothelial cells exposed to oxidative stress, with a particular focus on the role of autophagy. Endothelial EA.hy926 cells were employed as a model of the human endothelium, and tert-butyl hydroperoxide (t-BOOH) was used to induce oxidative stress. To evaluate the protective mechanisms, we analyzed the expression of autophagy-related proteins in OA-treated cells in the presence or absence of chloroquine (CQ), an inhibitor of autophagic flux. Moreover, we determined redox-related responses, including intracellular reactive oxygen species (ROS) production, glutathione (GSH) levels, and the activities of the antioxidant enzymes glutathione peroxidase (GPx) and glutathione reductase (GR).

## Experimental

### Materials and methods

#### Reagents

Tert-butyl hydroperoxide (t-BOOH), GR, reduced and oxidized (GSSG) glutathione, di-chlorofluorescin (DCFH), o-phthaldialdehyde (OPT), nicotine adenine dinucleotide phosphate reduced salt (NADPH), 2,4-dinitrophenylhydrazine (DNPH), 1,1,3,3-tetraethoxypropane (TEP), chloroquine (CQ), gentamicin, penicillin G, streptomycin, and sodium oleate were purchased from Sigma Chemical Co. (Madrid, Spain). Rapamycin was purchased from Merck (number 553,210). Acetonitrile, methanol of HPLC grade, dimethyl sulfoxide (DMSO) of analytical grade, and all other usual laboratory reagents were acquired from Panreac (Barcelona, Spain). The Bradford reagent was obtained from Bio-Rad Laboratories S.A. DMEM and fetal bovine serum (FBS) were obtained from Cultek (Madrid, Spain). All other reagents were of analytical quality.

#### Sample preparation

Fatty acid supplemented medium was prepared according to previously published protocols [19]. Oleic acid stock solutions of 200 mM were prepared in 100% EtOH. Working solutions of 1 mM OA were made by incubating the fatty acids in media containing 10% endotoxin and fatty acid-free BSA at 37 °C for 30–60 min with occasional vortexing. This solution was then added to the cells to obtain the final fatty acid concentrations. The OA/albumin molar ratio was maintained at < 3 to ensure that the fatty acid was bound to albumin, and equal volumes of the medium/EtOH/BSA vehicle were applied to control cells [20].

#### Cell culture

EA.hy926, a human hybrid cell line, was a kind gift from Profs. Patricio Aller and Carmelo Bernabéu, Centro de Investigaciones Biológicas, CSIC, Madrid, Spain. Cells were maintained in a humidified incubator containing 5% CO2 and 95% air at 37ºC and grown in DMEM high glucose (4,5 g/L) medium supplemented with 10% fetal bovine serum, 2 mM l-Glutamine and 50 mg/L of each of the following antibiotics: gentamicin, penicillin, and streptomycin. The culture medium was changed every other day in order to remove the non-adherent and dead cells, and the plates were usually split 1:3 when they reached confluence.

#### Cell treatment

1 mM stock solution of OA in serum-free culture medium was prepared and from these different concentrations of OA (10–100 µM) were added to the cell plates for 22 or 24 h to study the direct/basal effects of the compounds. To assay the protective effect of OA against an oxidative insult with 200 µM t-BOOH for 4 h, cells were pretreated with 25 µM OA for 20 h and then, cell plates were washed out a new media containing the pro-oxidant t-BOOH for 4 h.

#### Evaluation of cell viability

Cell viability was determined via a crystal violet assay [21]. The cells were seeded at low density (1 × 10^4^ cells per well) in 96-well plates, grown for 22 h and incubated with crystal violet (0.2% in ethanol) for 20 min. The plates were rinsed with water, and 1% sodium dodecyl sulfate was added. The absorbance of each well was measured at 570 nm via a microplate reader.

#### Determination of ROS

Cellular ROS were quantified via the DCFH assay via a microplate reader. For the assay, the cells were seeded in 24-well plates at a density of 2 × 10^5^ cells per well and conditions added. Prior to the end of the assay, 5 µM DCFH was added to the wells for 30 min at 37 °C. Then, the cells were washed twice with serum-free medium before the multiwell plates were measured in a fluorescence microplate reader at an excitation wavelength of 485 nm and an emission wavelength of 530 nm. Intracellular oxidants oxidize DCFH to dichlorofluorescein (DCF) that emits fluorescence that is quantified fluorescence over a period of 90–120 min, producing a reasonable estimation of the degree of cellular oxidative stress through the overall oxygen species generated under the different conditions. The assay has been described elsewhere [22, 23].

#### Western blotting

EA.hy926 cells were washed with ice-cold PBS and then lysed in buffer containing 1% (v/v) Nonidet P40, 50 mM Tris/HCl, 5 mM EDTA, 5 mM EGTA, 150 mM NaCl, 20 mM NaF, 1 mM phenylmethylsulfonyl fluoride, 10 µg/mL aprotinin, and 2 µg/mL leupeptin (pH 7.5). Cellular debris was pelleted by centrifugation at 15,000 × g for 15 min at 4 °C, and the resulting supernatants were collected for protein determination. The samples were submitted to SDS‒PAGE (8–15% gels), followed by Western blotting and visualization via an enhanced chemiluminescence (ECL) Western blotting detection kit (GE Healthcare Bio-Sciences; Madrid, Spain; RPN2106). Densitometric quantification of the blots was performed with NIH ImageJ (https://imagej.nih.gov/ij/; access on 15 June 2021). We used rapamycin and chloroquine as positive and negative controls, respectively, for autophagy. For the performance of these experiments, we considered the published Autophagy Guideline [24].

#### Antibodies

The anti-ULK1 #8054, anti-phospho-ULK1 (Ser555) #5869, anti-p70 #9202, anti-phospho-p70 (Thr389) #9205, and anti-LC3B #4108 antibodies were obtained from Cell Signaling Technology (Beverly, MA, USA), and the anti-(GP62-C) p62/SQSTM1 (C-terminus) antibody was obtained from Progen. An anti-β-actin antibody (A5316) from Sigma‒Aldrich was used. The secondary HRP-conjugated antibodies used, anti-rabbit (NA934) and anti-mouse (NA931), were obtained from GE Lifesciences (Marlborough, MA).

#### Determination of the GSH concentration

The content of GSH was quantified via the fluorometric assay [25] with slight modifications. The method is based on the reaction of GSH with OPT at pH 8.0, and the fact that OPT reacts not only with GSH but also with other thiols, such as cysteine and N-acetylcysteine, was overcome by comparison to appropriate controls allowing a reliable estimation. After the different treatments, the culture medium was removed, and the cells (4 × 10^6^) were detached and homogenized via ultrasonication with 5% trichloroacetic acid containing 2 mM EDTA. Following the centrifugation of the cells for 30 min at 1000 × g, 50 µL of the clear supernatant was transferred to a 96-well plate for the assay. The fluorescence was measured at an excitation wavelength of 345 nm and an emission wavelength of 425 nm. The results of the samples were compared with those of a standard curve of GSH.

#### Determination of GPx and GR activity

For the assay of GPx and GR activity, treated cells (4 × 10^6^) were suspended in PBS and centrifuged at 300 × g for 5 min to pellet the cells. The cell pellets were resolved in 20 mM Tris, 5 mM EDTA, and 0.5 mM mercaptoethanol, submitted to sonication and centrifuged at 3000 × g for 15 min. Supernatants were used to analyze enzyme activities. GR activity was determined by the decrease in absorbance due to the oxidation of NADPH utilized in the reduction of oxidized glutathione [22, 23]. The determination of GPx activity is based on the oxidation of GSH by GPx, which uses t-BOOH as a substrate, coupled with the disappearance of NADPH by GR, as described above [23] with slight modifications. The protein concentration was measured via the Bradford reagent.

### Statistical analysis

The statistical analysis of the data was as follows: Prior to analysis, the data were tested for homogeneity of variance by the Levene test; for multiple comparisons, one-way ANOVA was followed by a Bonferroni correction when the variances were homogeneous or by the Tamhane test when the variances were not homogeneous. The level of significance was *p* < 0.05. SPSS version 23.0 was used. Different small letters above the data bars or as superscripts indicate statistically significant differences among the different groups or conditions; the data that share a statistical letter are not significantly different.

## Results and discussion

### Dose response of OA on cell viability

**Fig. 1:**
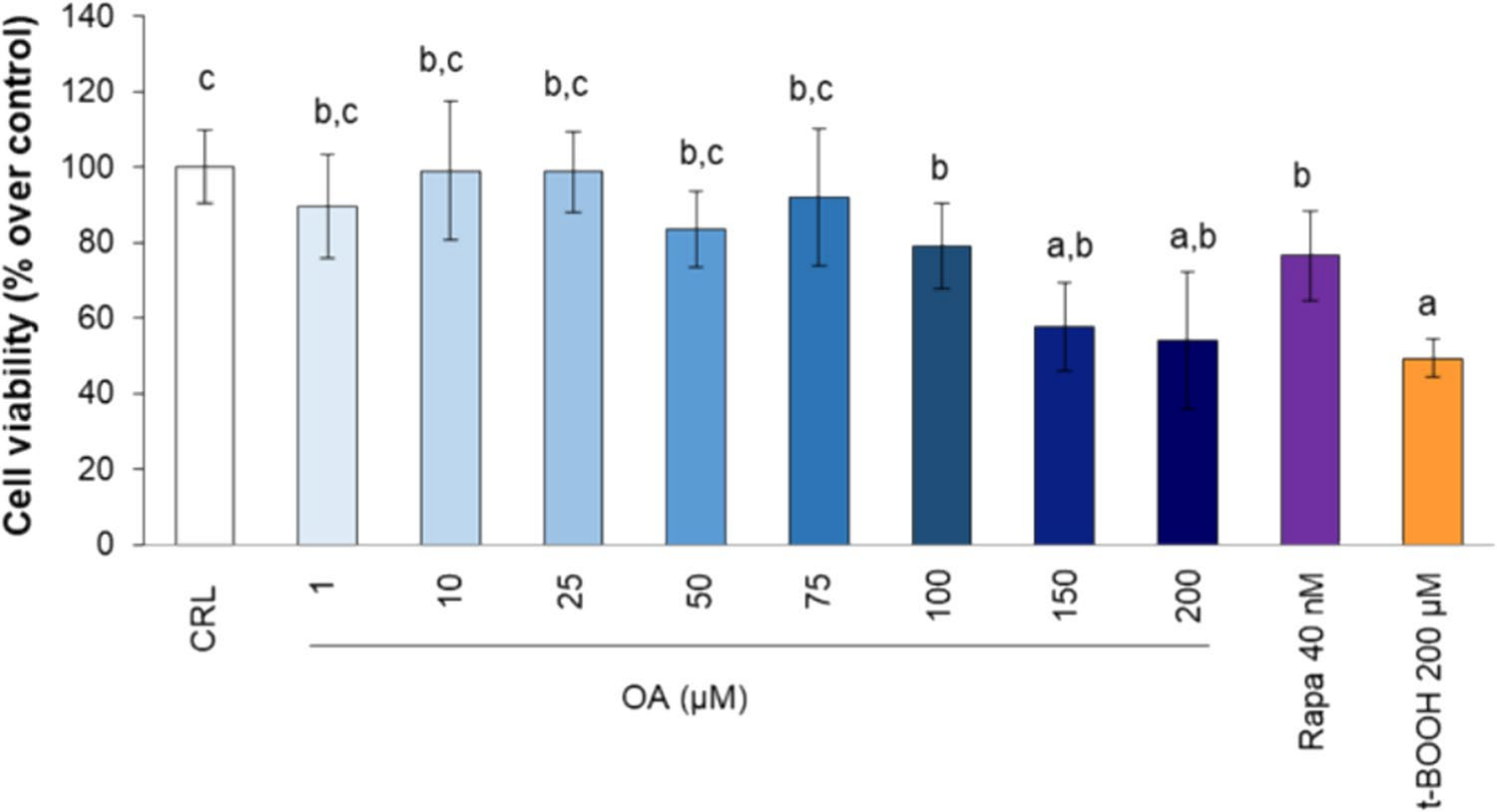
Direct effects of oleic acid (OA) and rapamycin (Rapa) on EA.hy926 cell viability. t-BOOH (200 µM) was used as a toxic insult. The results are presented as the means ± SDs (*n* = 8 replicates). Different letters above the data bars indicate significant differences (*p* < 0.05) among the data, whereas the data that show or share the same letters are not significantly different and should be considered equal

Viability of cultured EA.hy926 cells for 22 h was tested in the presence of a wide range of doses from 1 to 200 µM OA, and the results of cell are depicted in Fig. 1. OA at concentrations up to 100 µM did not significantly affect the number of cells, but a significant decrease was observed in the plates of cells treated with 100–200 µM OA, indicating dose-dependent cytotoxicity. As a positive control for cell damage and mortality, cells were treated with 200 µM t-BOOH for 4 h, and the results revealed a 50% decrease in cell viability, clearly indicating that oxidative stress-induced cell death. A rate of cell mortality similar to that of cells treated with 50–100 µM OA was also observed in cells treated with 40 nM rapamycin (Fig. 1). Thus, OA at concentrations greater than 75 µM induced cell death in cultured endothelial EA.hy926 cells.

### Dose response of OA to ROS levels

**Fig. 2:**
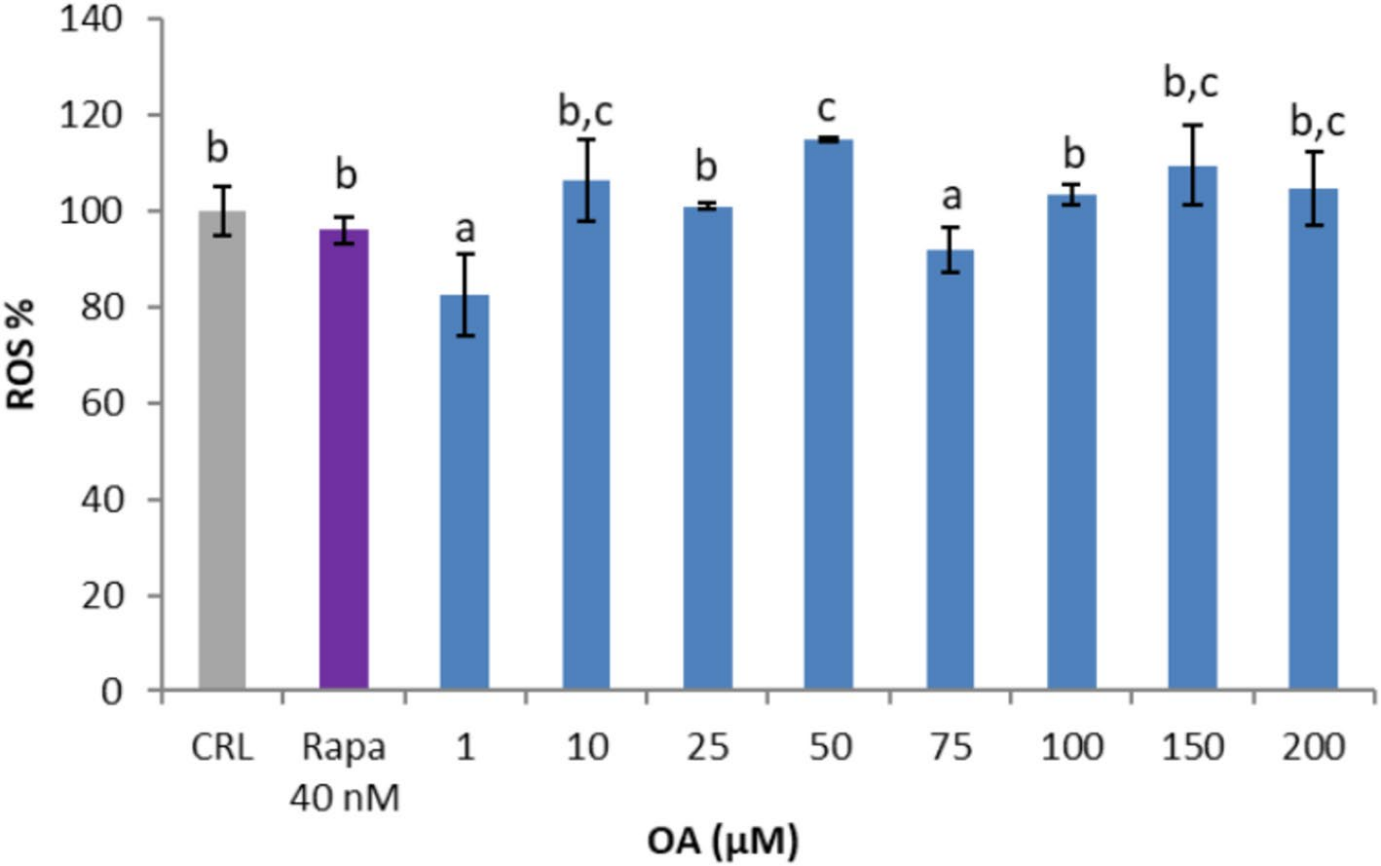
Direct effects of oleic acid (OA) and rapamycin (Rapa) on the production of ROS by EA.hy926 cells. Different letters above the data bars indicate significant differences (p < 0.05) among the data, whereas the data that show or share the same letters are not significantly different and should be considered equal

Although we previously reported that OA concentrations in the low µM range (0.1–5 µM) did not evoke changes in ROS production in EA.hy926 cells [11], we needed to ensure that the tenfold higher doses tested in this study did not significantly affect the redox status of the cultured endothelial cells. Thus, a wide range of eight OA concentrations ranging from 1 to 200 µM were tested under basal conditions for 24 h, and ROS generation was determined. Figure 2 shows that a slight but significant decrease in ROS was observed in cells treated with 1 µM and 75 µM OA, as well as with rapamycin. However, a slight but significant increase in ROS generation was detected in cells treated with 50 µM OA. Overall, direct treatment of endothelial cells with OA concentrations above the physiological range of up to 75 µM for 24 h did not evoke alterations in the concentration of ROS, which seemed relevant for affecting the physiological condition of the cells. In contrast, concentrations of and above 100 µM evoked slight but apparent increases in ROS, which could be the cause of the reduced cell viability observed under these conditions (Fig. 2).

### OA stimulates autophagy

**Fig. 3:**
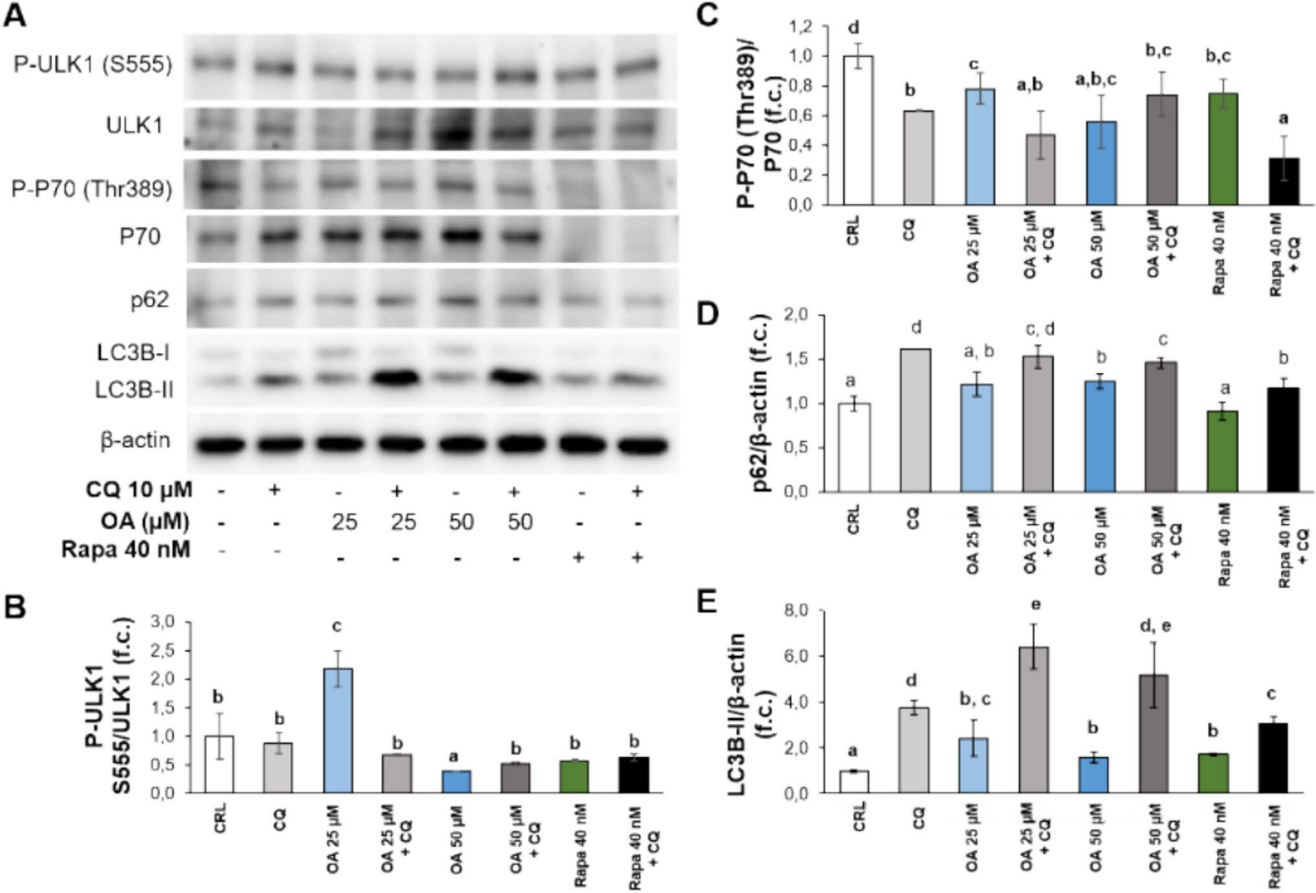
Direct effects of treatment with oleic acid (OA) with or without chloroquine (CQ) or rapamycin (Rapa) on autophagy activity in EA.hy926 cells. Representative blots (A) and quantification of the results (B-E) of the phosphorylation status of ULK1 (Ser555) and P70 (Thr308), two of the main targets of AMPK and mTORC1, respectively, as well as the expression levels of autophagy-related proteins (p62 and LC3B). Within each panel, different letters above the data bars indicate significant differences (*p* < 0.05) among the data, whereas the data that show or share the same letters are not significantly different and should be considered equal (*n* = 3)

To analyze the role of OA in the modulation of autophagy, EA.hy926 cells were subjected to 25 or 50 µM OA for 24 h in the presence (or absence) of 10 µM chloroquine (CQ) for the last 18 h. CQ was used to inhibit autophagy flux by decreasing the fusion between autophagosomes and lysosomes. In the presence of CQ, the levels of p62 and LC3B-II increased, which indicates functional autophagic flux in these cells [26]. As a positive control of autophagy activation, cells were treated with 40 nM rapamycin (or rapa for short) for 24 h. Compared with control cells, cells stimulated with 25 µM OA presented significantly increased phosphorylation levels of ULK1 (at Ser555), suggesting the activation of AMPK signaling activity. In parallel, OA (25 or 50 µM) and rapa significantly decreased the phosphorylation of p70 (at Thr389), indicating the inhibition of mTORC1 activity (Fig. 3). Additionally, p62 expression was significantly greater in cells stimulated with OA or rapa and CQ than in those stimulated with the same treatments in the absence of CQ. A significant increase in the ratio of the lipidated form of LC3B (LC3B-II) was also detected when the cells were stimulated with 25 or 50 µM OA or rapa, and the ratio further increased in the presence of CQ (Fig. 3). Overall, these results demonstrated that 25 or 50 µM OA activated autophagy in EA.hy926 cells to a similar or greater extent than rapamycin did.

### Protective effect of OA on ROS production

**Fig. 4:**
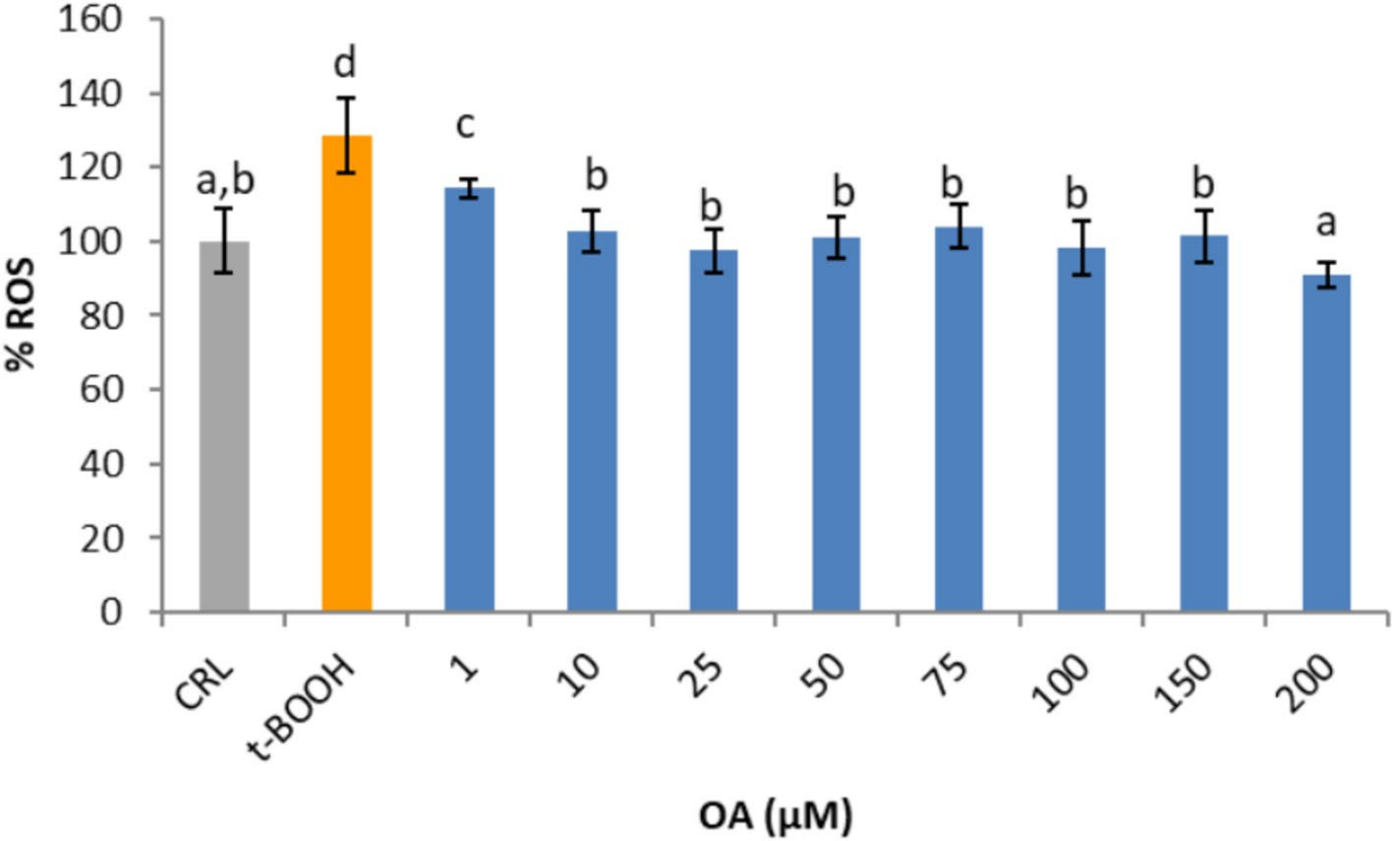
Effect of pretreatment with oleic acid (OA) on ROS production in oxidative stress-induced EA.hy926 cells. The results are presented as the means ± SDs (*n* = 4 replicates). Different letters above the data bars indicate significant differences (*p* < 0.05) among the data, whereas the data that show or share the same letters are not significantly different and should be considered equal

Since there was a clear differential effect of OA on ROS production and cell viability depending on the concentration, the next step was to test whether all the tested concentrations of OA were capable of protecting endothelial cells against a chemically induced oxidative challenge. To that end, cells were subjected to a pro-oxidant agent such as t-BOOH, which decomposes to peroxyl radicals and generates lipid peroxides and ROS. As a clear indication of a situation of oxidative stress, a significant 30% increase in the intracellular ROS concentration was observed when EA.hy926 cells were treated with 200 µM t-BOOH for 4 h (Fig. 4). However, this increase in ROS levels was significantly reduced when endothelial cells were pretreated for 20 h prior to stress with any OA concentration. Additionally, there was a statistically significant dose-dependent reduction in ROS production at doses of 1 µM, intermediate doses, and then 200 µM. Interestingly, doses of OA that directly increased (50 µM) or tended to increase (150 and 200 µM) ROS also evoked a protective reduction in the reactive species, suggesting opposite effects under basal and oxidative conditions. Despite this curious response of EA.hy926 cells to OA under conditions of oxidative stress, to avoid the potential deleterious effects of OA at concentrations higher than 25 µM on endothelial function and remain the closest as possible to physiological conditions, a concentration of 25 µM was selected as a chemopreventive dose for the rest of the experiments. In addition, this dose of 25 µM OA was the minimal concentration that increased LC3B-II and activated autophagy, which is necessary to elucidate the potential contribution of this process to the chemoprotective mechanism.

### Role of OA-mediated activation of autophagy in protection against oxidative stress

**Fig. 5:**
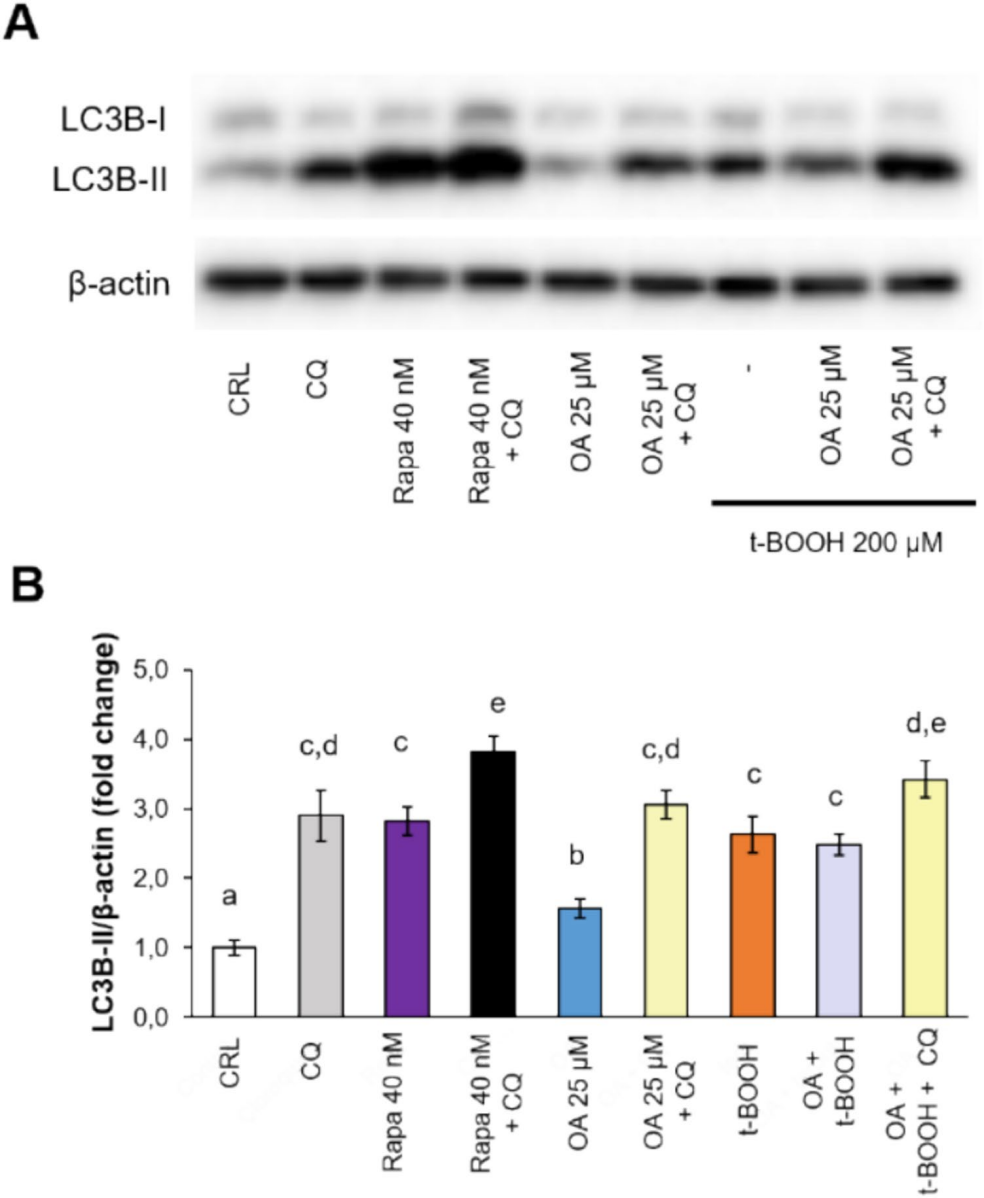
Effect of oleic acid (OA) treatment on LC3B protein levels in EA.hy926 cells under t-BOOH-mediated oxidative stress. The cells were treated with 10 µM chloroquine (CQ), alone or in combination with 25 µM OA for 20 h, followed by stimulation with or without 200 µM t-BOOH for 4 h. Rapamycin (Rapa) was used as a positive control for autophagy activation. Representative blots (A) and quantification of the results (B) of the protein expression levels of LC3B. The results are presented as the means ± SDs (*n* = 4 replicates). Within each panel, different letters above the data bars indicate significant differences (*p* < 0.05) among the data, whereas the data that show or share the same letters are not significantly different and should be considered equal

EA.hy926 cells were pretreated with 25 µM OA in the presence or absence of 10 µM CQ for 20 h. Subsequently, the cells were exposed to an oxidative stressor (200 µM t-BOOH for 4 h). Compared with control-treated cells, OA- or rapa-treated cells and CQ-treated cells presented significantly increased LC3-II levels in the absence of CQ, confirming that OA activates autophagy in these cells (Fig. 5). When an oxidative stressor (t-BOOH) was used, the LC3B-II protein levels increased, indicating that autophagy was activated. Compared with t-BOOH alone, 25 µM OA did not further increase the level of LC3-II in cells subjected to this oxidative insult. However, in cells treated with OA and t-BOOH, stimulation with CQ significantly increased LC3-II levels (Fig. 5), indicating that OA was able to effectively activate autophagy as a cellular defense mechanism under stressful conditions.

### Role of autophagy in ROS production

**Fig. 6:**
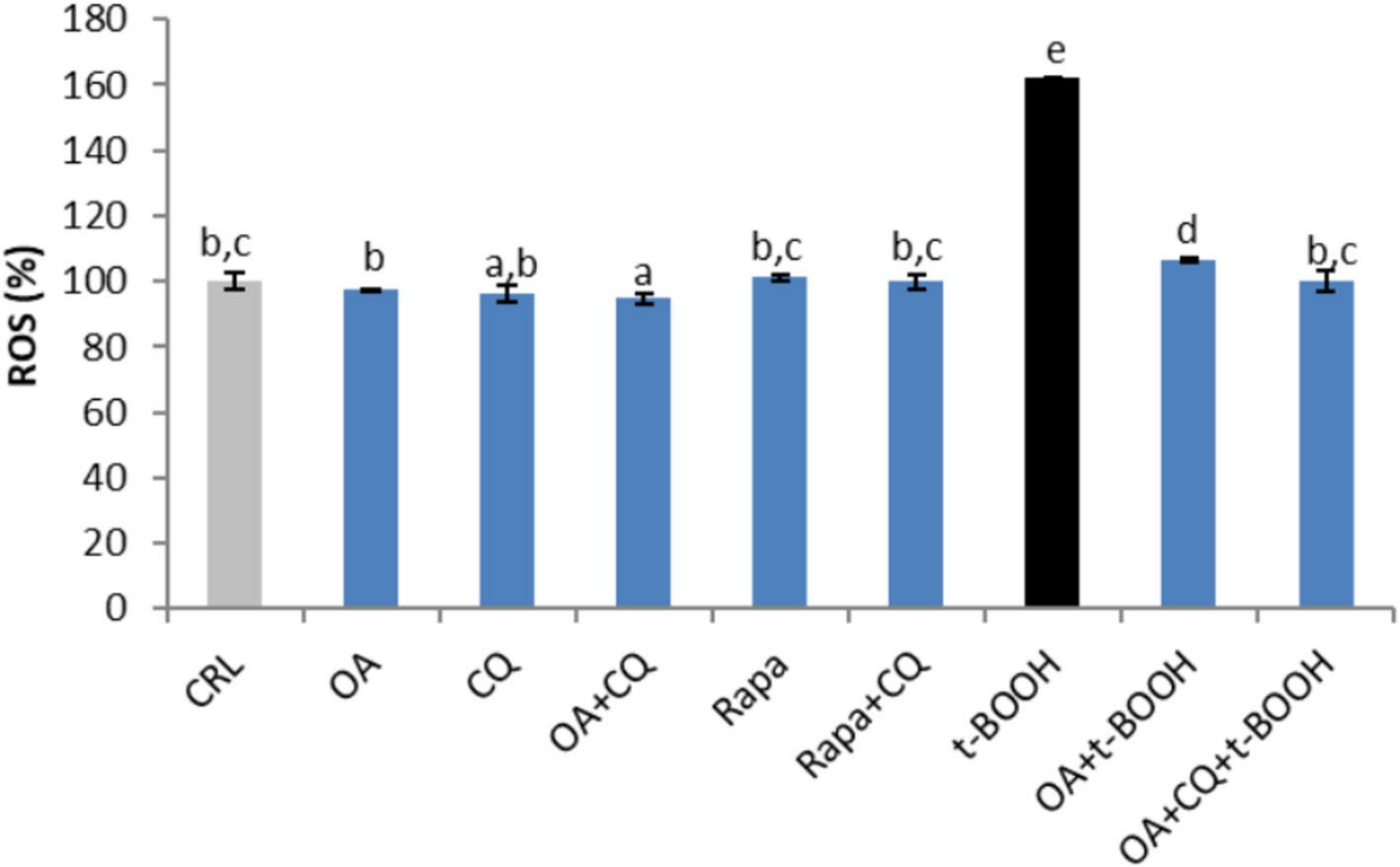
Effect of pretreatment with 25 µM oleic acid (OA) on ROS production by EA.hy926 cells. The cells were treated with 10 µM chloroquine (CQ), alone or plus 25 µM OA, as well as 40 nM rapamycin (rapa), alone or plus CQ. To induce oxidative stress, the cells were stimulated with 200 µM t-BOOH (4 h) in the absence or presence of OA or OA plus CQ. The results are presented as the means ± SDs (*n* = 4 replicates). Different letters above the data bars indicate significant differences (*p* < 0.05) among the data, whereas the data that show or share the same letters are not significantly different and should be considered equal

To determine whether the effect of OA on autophagy in EA.hy926 cells was involved in the protective mechanism of fatty acids against oxidative stress-induced ROS, the effect of CQ was tested. As depicted in Fig. 6, treatment of cells with 10 µM chloroquine, alone or plus 25 µM OA, or 40 nM rapamycin, alone or plus CQ, did not significantly affect ROS production. As expected, the substantial increase in ROS in cells treated with 200 µM t-BOOH for 4 h was significantly reduced by pretreatment with 25 µM OA for 20 h, and the inhibition of autophagy by the addition of 10 µM CQ slightly but significantly decreased the level of ROS induced by t-BOOH. However, this significant reduction in ROS may not be physiologically relevant. These data indicate that the induction of autophagy by OA is not involved in the reduction in oxidative stress mediated by t-BOOH.

### Effect of autophagy on the GSH concentration

**Fig. 7:**
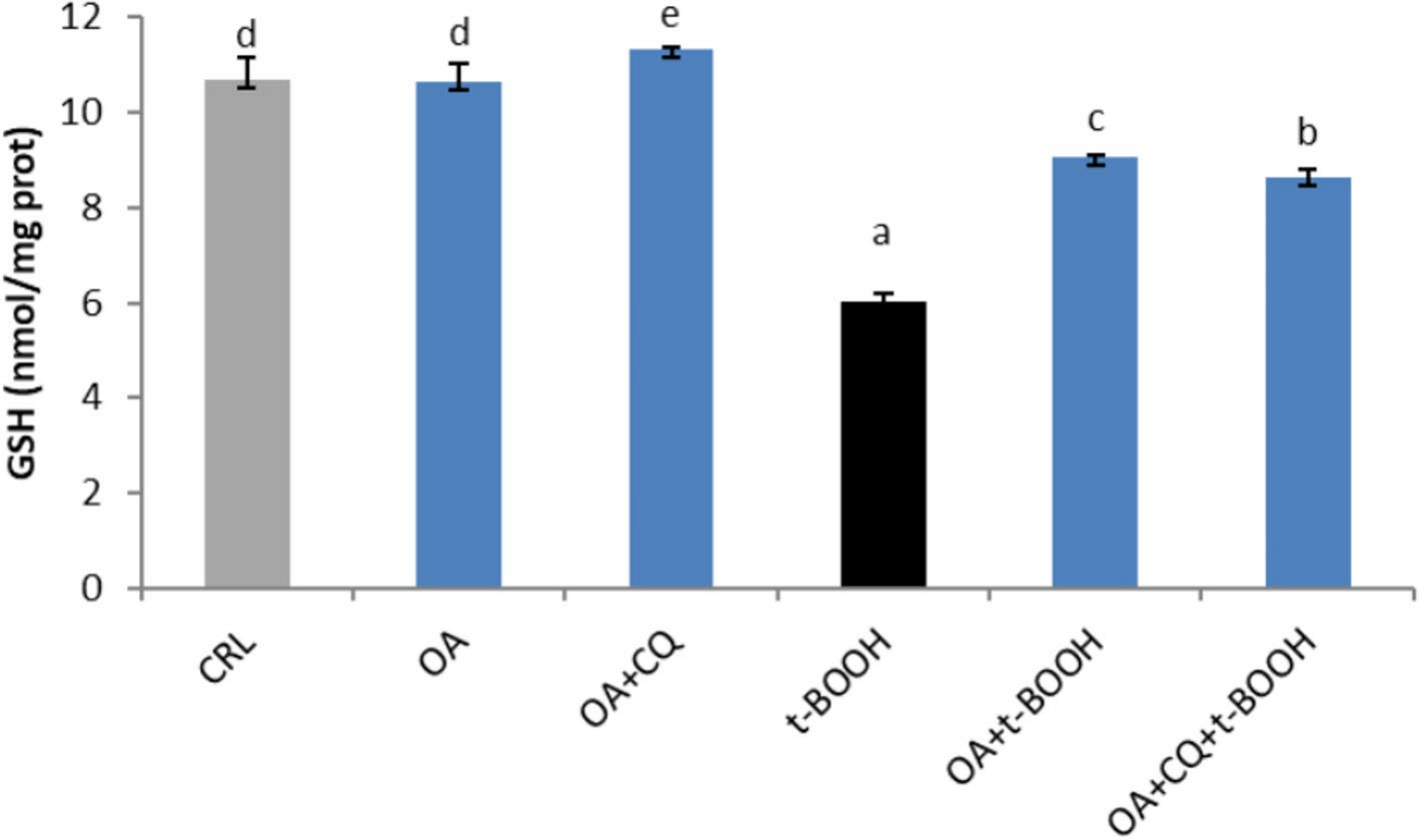
Effect of 25 µM oleic acid (OA) on the GSH concentration in EA.hy926 cells. The cells were cotreated with 25 µM OA alone or OA plus 10 µM chloroquine (CQ), 25 µM OA plus 200 µM t-BOOH, or 25 µM OA plus 10 µM CQ plus 200 µM t-BOOH for 22 h. The results are presented as the means ± SDs (*n* = 4 replicates). Different letters above the data bars indicate significant differences (*p* < 0.05) among the data, whereas the data that show or share the same letters are not significantly different and should be considered equal

To test the potential role of autophagy in the protective effect of OA on GSH under conditions of oxidative insult, endothelial cells were treated with OA alone or in combination with CQ prior to being treated with t-BOOH. Figure 7 shows that treatment of cells with OA evoked no significant changes in the basal values of GSH, whereas in the presence of CQ, OA slightly but significantly increased the basal values of GSH. Conversely, treatment with 200 µM t-BOOH for 4 h provoked a 50% decrease in GSH, which was partly but significantly reversed by 25 µM OA. Under oxidative stress conditions, autophagy inhibition caused a slight but significant decrease in the recovery of GSH levels mediated by OA (Fig. 7). These results indicate that autophagy has a minor but appreciable effect on the GSH response of EA.hy926 cells to oxidative stress in the presence of OA.

### Effect of autophagy on GPx and GR activities

**Fig. 8:**
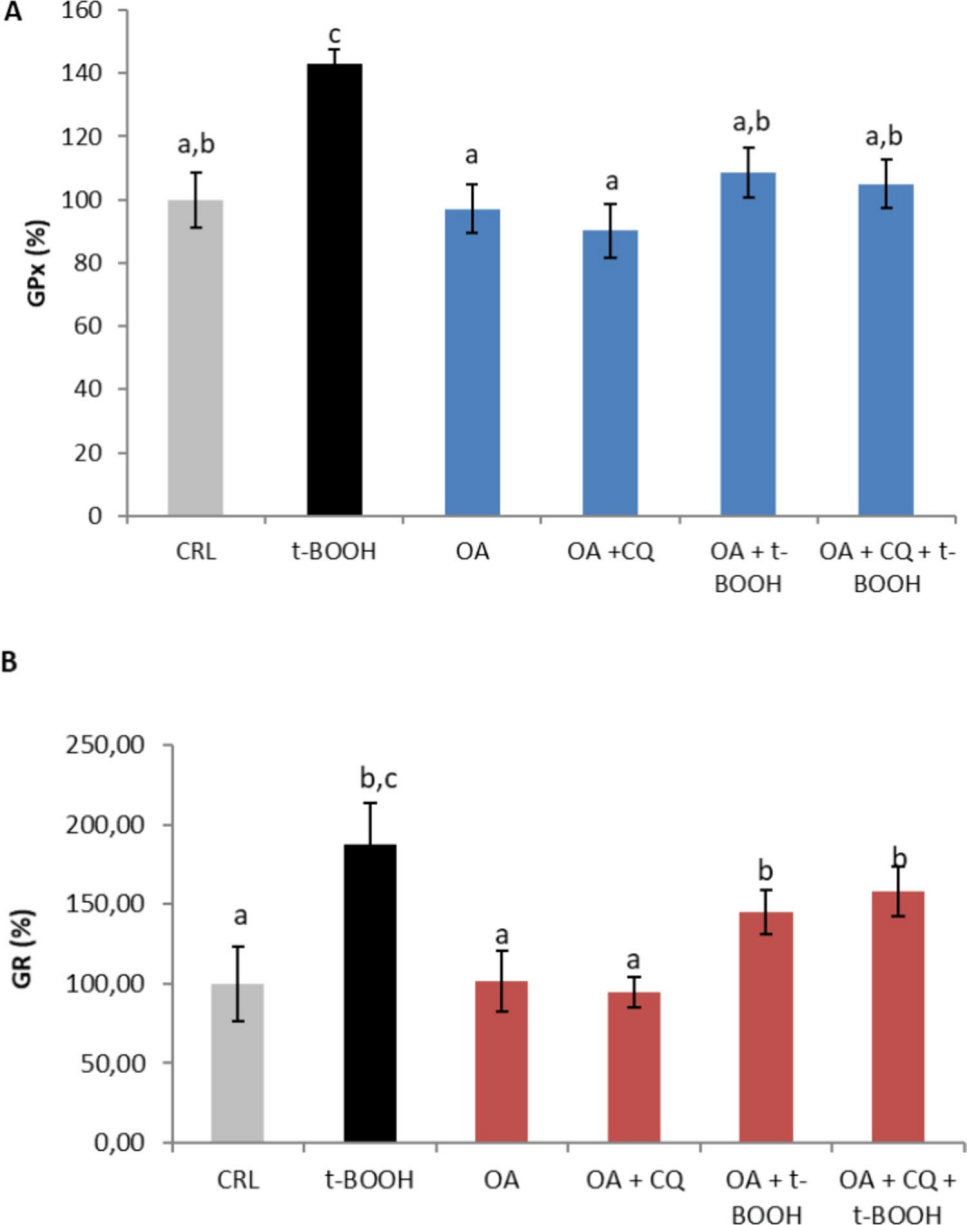
Effect of oleic acid (OA) on a) GPx activity and b) GR activity in EA.hy926 cells. The cells were treated with 25 µM OA alone or OA plus 10 µM chloroquine (CQ), 25 µM OA plus 200 µM t-BOOH, or 25 µM OA plus 10 µM CQ plus 200 µM t-BOOH for 22 h. The results are presented as the means ± SDs (*n* = 4 replicates). Within each panel, different letters above the data bars indicate significant differences (*p* < 0.05) among the data, whereas the data that show or share the same letters are not significantly different and should be considered equal

To study the role of autophagy on the protective capacity of antioxidant enzymes against oxidative stress in cultured endothelial cells in OA, EA.hy926 cells were subjected to a severe oxidative challenge, and the effect of CQ on the chemoprotective antioxidant enzyme defenses of OA was investigated. Figure 8 shows that both 25 µM OA alone and OA plus 10 µM CQ did not affect the steady-state activity of either the antioxidant enzyme GPx (panel A) or GR (panel B). The activity of both enzymes significantly increased in response to the stressful insult induced by t-BOOH, and similar partial protection was observed in both enzyme activities when the cells were treated with OA compared with those when they were treated with OA plus CQ, indicating that CQ has no effect on the protective effect of OA.

### Effect of chloroquine on the protection of cell viability by OA

**Fig. 9:**
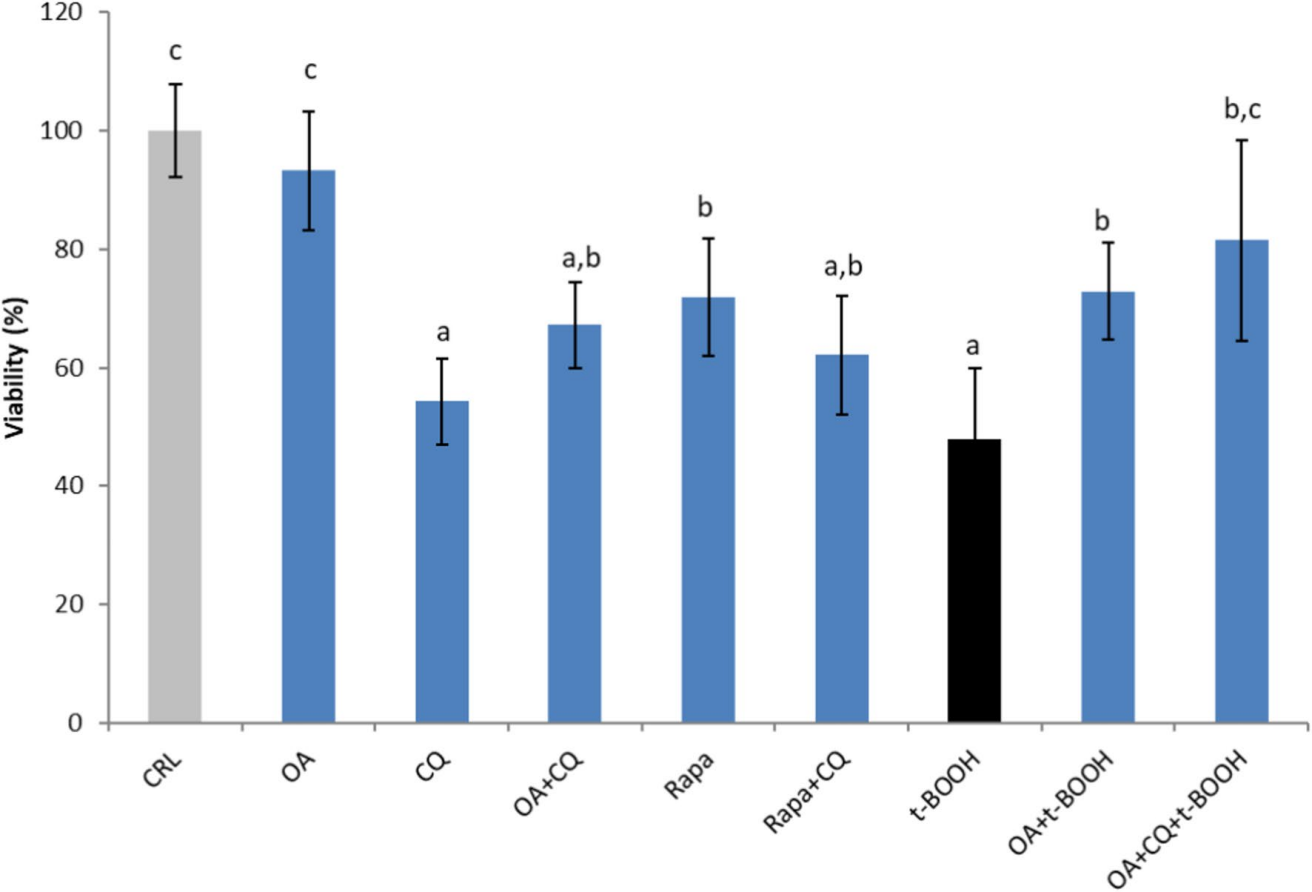
Direct effects of 25 µM oleic acid (OA), 10 µM chloroquine (CQ) and 40 nM rapamycin (rapa), alone or in combination, on EA.hy926 cell viability. t-BOOH (100 µM) was used as a toxic insult. The results are presented as the means ± SDs (n = 8 replicates). Different letters above the data bars indicate significant differences (*p* < 0.05) among the data, whereas the data that show or share the same letters are not significantly different and should be considered equal

Finally, to elucidate the potential contribution of autophagy to the protective effect of OA on endothelial cell viability, EA.hy926 cells were subjected to oxidative stress and protected from OA in the presence or absence of CQ. Figure 9 shows that inhibition of autophagy with CQ considerably decreased endothelial cell viability to a similar extent as t-BOOH did, suggesting the requirement of the pathway for cell survival. The dramatic decrease in cell viability of approximately 50% evoked by t-BOOH was substantially reversed in cells treated with 25 µM OA, and further addition of CQ did not significantly affect recovery.

## Discussion

The main goal of the present study was to evaluate whether autophagy is potentially involved in the protective effect of OA against oxidative stress in endothelial cells. Since the activation of autophagy may play a protective role in stressed cells [12–16], it seems very attractive to study whether this process is also involved in the protective mechanism of OA previously shown in stressed endothelial cells [11]. The first step was to discern whether OA directly induced autophagy in endothelial cells, and EA.hy926 cells displayed a significant increase in autophagy when treated with fatty acids, a finding previously unreported. By using specific regulators of autophagy, the role of this process was subsequently studied in cells subjected to severe conditions of oxidative stress.

Oxidative stress, a dominance of oxidants over antioxidants and repair processes provoking a disruption of redox signaling and molecular damage, has proven to be an intrinsic pathogenic mechanism connecting elevated serum fatty acids, endothelial dysfunction, and cardiovascular disease [20, 27, 28]. Thus, the inhibition or attenuation of pro-oxidant conditions in endothelial cells might be an effective strategy to prevent or restrain vascular dysfunction [2, 3, 29, 30]. Although the maintenance of redox balance is dependent mainly on cell antioxidant defenses, other adaptive response mechanisms might be involved and essential for homeostatic control, i.e., mitogen-activated protein kinases [31], apoptosis [32], and autophagy [15, 16, 33]. Indeed, attention is increasing on the study of the role of autophagy since it has been proposed as a target for both neurodegenerative diseases [16] and cancer [15]. In this regard, the measurable contribution of autophagy to the protective effect of antioxidants has been previously reported [12–14].

EA.hy926, an established cell line derived from human umbilical vein endothelial cells that has been extensively used as a reliable model of the vascular endothelium to test the effects of natural products [11, 32, 34, 35], and to investigate the potential role of autophagy in the protective effect of OA on the endothelium, was used for this study. In a previous study from our laboratory, a selection of OA doses in the low-µM range conferred significant protection against the oxidative stress induced by EA.hy926, which was similar to the results of the present study [11]. However, since the set of OA concentrations tested in that study was not able to activate the autophagy pathway, we increased the OA dose to a sufficient range to stimulate autophagy and elucidate its potential contribution to the overall protective process. The results demonstrated that the activation of autophagy occurred (as assessed by the increase in the LC3B-II protein levels) when the cells were treated with OA at 25 and 50 µM. Compared with nontreated cells, cells treated with CQ presented increased LC3B-II protein levels in the presence of 25 µM OA, indicating that efficient enhancement of autophagic flux occurs. However, in an acute pro-oxidative situation, the activation of autophagy seems to have a very limited effect on the chemoprotective mechanism of OA in cultured endothelial cells.

EA.hy926 viability was not altered by treatment with OA at concentrations up to 75 µM for 22 h, indicating that no endothelial cell toxicity occurred under basal conditions. It is mandatory to ensure that no direct cell damage is caused since, as stated above and in many other studies [8–10, 27, 30], elevated doses of FFAs may also act as pro-oxidants/proinflammatory agents in the endothelium and evoke vascular damage. According to the autophagy data, OA doses of 25–50 µM were necessary to reduce mTORC1 activity and activate autophagy in cultured EA.hy926 cells; consequently, considering that 50 µM OA induced a slight reduction in cell viability and to stay as close as possible to the physiological range, 25 µM OA seems to be the most appropriate concentration to reveal the possible contributory role of autophagy. The OA dose selected in the present study is still well above the nanomolar levels usually found in postprandial blood but is still far from the systemic levels necessary to evoke endothelial damage and increase the atherogenic index [36].

Different markers of redox status and antioxidant defenses are necessary for an efficient protective mechanism and the putative involvement of autophagy. Thus, the considerable increase in ROS in cells stressed with t-BOOH was substantially reversed by 25 µM OA, and the addition of chloroquine further decreased the level of ROS induced by t-BOOH, ruling out a potential synergistic chemoprotective effect. A somehow different result was obtained when the GSH concentration was evaluated; the dramatic depletion of GSH in t-BOOH-stressed cells was significantly prevented in part by OA, but the degree of recovery of GSH was slightly reduced when autophagy was abolished by CQ. Furthermore, no significant change in the protective response of the antioxidant defense enzymes GPx and GR was found when chloroquine was supplemented with OA in stressed endothelial cells, suggesting a limited contribution of autophagy to the chemoprotective response of the antioxidant defense system. These results are not the same as those reported with curcumin in EA.hy926 cells [12] or with *Sambucus nigra* fruit extract in oral dysplastic cells [13], where autophagy seemed to play a major contributory role. However, this discrepancy in the effect of autophagy might be explained by the differences in the experimental conditions of the mentioned reports, where chloroquine was not used in the experiments. This result was also unexpected because, in previous reports, treatment of cells with antioxidant compounds or antioxidant-rich extracts might suggest that recovery of the antioxidant defenses was sufficient to improve cell survival; however, OA has no antioxidant capacity and might require other synergic mechanisms to address the oxidative insult.

## Conclusions

The aim of this study was to assess the contribution of autophagy to the protective effect of OA on endothelial cell viability under conditions of oxidative stress. The effect of chloroquine under steady-state conditions was promising considering that there was decreased viability in EA.hy926 cells treated with only the drug, suggesting the necessity of functional autophagy for endothelial cell growth and survival. Despite these positive results, we found no significant decrease in the capacity/activity of most markers of the oxidative defense system or in cell survival when autophagy was inhibited. These results do not completely rule out the implication of this mechanism in the protection exerted by OA but strongly suggest a limited contribution under conditions of severe oxidative stress, such as that established in this study in endothelial cells.

## Supplementary Information

Below is the link to the electronic supplementary material.

Supplementary file1 (PPTX 5075 kb)

## Data Availability

All the data supporting this article have been included as part of the manuscript.

## References

[1] Chistiakov DA, Orekhov AN, Bobryshev YV (2015) Endothelial barrier and its abnormalities in cardiovascular disease. Front Physiol 6:365–375. 10.3389/fphys.2015.00365 26696899 10.3389/fphys.2015.00365 PMC4673665

[2] Massaro M, Scoditti E, Carluccio MA, de Caterina R (2019) Oxidative stress and vascular stiffness in hypertension: a renewed interest for antioxidant therapies? Vasc Pharmacol 116:45–50. 10.1016/j.vph.2019.03.004 10.1016/j.vph.2019.03.004 30946986

[3] Senoner T, Dichtl W (2019) Oxidative stress in cardiovascular diseases: still a therapeutic target? Nutrients 11:2090. 10.3390/nu11092090 31487802 10.3390/nu11092090 PMC6769522

[4] Sies H, Belousov VV, Chandel NS, Davies MI, Jones DP, Mann GE, Murphy MP, Yamamoto M, Winterbourn C (2022) Defining roles of specific reactive oxygen species (ROS) in cell biology and physiology. Nat Rev Mol Cell Biol 23:499–515. 10.1038/s41580-022-00456-z 35190722 10.1038/s41580-022-00456-z

[5] Shaito A, Aramouni K, Assaf R, Parenti A, Orekhov A, Yazbi AE, Pintus G, Eid AH (2022) Oxidative stress-induced endothelial dysfunction in cardiovascular diseases. Front Biosci (Landmark Ed) 27(3):105. 10.31083/j.fbl2703105 35345337 10.31083/j.fbl2703105

[6] Nemecz M, Constantin A, Dumitrescu M, Alexandru N, Filippi A, Tanko G, Georgescu A (2019) The distinct effects of palmitic and oleic acid on pancreatic beta cell function: the elucidation of associated mechanisms and effector molecules. Front Pharmacol 9:1554–1569. 10.3389/fphar.2018.01554 30719005 10.3389/fphar.2018.01554 PMC6348268

[7] Lundman P, Tornvall P, Nilsson L, Pernow J (2001) A triglyceride rich fat emulsion and free fatty acids, but not very low density lipoproteins, impair endothelium -dependent vasorelaxation. Atherosclerosis 159:35–41. 10.1016/s0021-9150(01)00478-6 11689204 10.1016/s0021-9150(01)00478-6

[8] Couloubaly S, Delomenie C, Rousseau D, Paul JL, Grynberg A, Pourci ML (2007) Fatty acid incorporation in endothelial cells and effects on endothelial nitric oxide synthase. Eur J Clin Investig 37:692–699. 10.1111/j.1365-2362.2007.01843.x 17696958 10.1111/j.1365-2362.2007.01843.x

[9] Lamers D, Schlich R, Greulich S, Sasson S, Sell H, Eckel J (2011) Oleic acid and adipokines synergize in inducing proliferation and inflammatory signaling in human vascular smooth muscle cells. J Cell Mol Med 15:1177–1188. 10.1111/j.1582-4934.2010.01099.x 20518853 10.1111/j.1582-4934.2010.01099.x PMC3822630

[10] Gremmels H, Bevers LM, Fledderus JO, Braam B, van Zonneveld AJ, Verhaar MC, Joles JA (2015) Oleic acid increases mitochondrial reactive oxygen species production and decreases endothelial nitric oxide synthase activity in cultured endothelial cells. Eur J Pharmacol 751:67–72. 10.1016/j.ejphar.2015.01.005 25595727 10.1016/j.ejphar.2015.01.005

[11] Palomino OM, Giordani V, Chowen J, Alfonso SF, Goya L (2022) Physiological doses of oleic and palmitic acids protect human endothelial cells from oxidative stress. Molecules 27:5217. 10.3390/molecules27165217 36014457 10.3390/molecules27165217 PMC9415781

[12] Guo S, Long M, Li X, Zhu S, Zhang M, Yang Z (2016) Curcumin activates autophagy and attenuates oxidative damage in EA.hy926 cells via the Akt/mTOR pathway. Mol Med Rep 13:2187–2193. 10.3892/mmr.2016.4796 26781771 10.3892/mmr.2016.4796

[13] Filip GA, Florea A, Olteanu D, Clichici S, David L, Moldovan B, Cenariu M, Scrobota I, Potara M, Baldea I (2021) Biosynthesis of silver nanoparticles using *Sambucus nigra* L. fruit extract for targeting cell death in oral dysplastic cells. Mater Sci Eng C 123:111974. 10.1016/j.msec.2021.111974 10.1016/j.msec.2021.111974 33812602

[14] Palomino O, García-Aguilar A, González A, Guillén C, Benito M, Goya L (2021) Biological actions and molecular mechanisms of *Sambucus nigra* L. in neurodegeneration: a cell culture approach. Molecules 26:4829. 10.3390/molecules26164829 34443417 10.3390/molecules26164829 PMC8399386

[15] Levy JMM, Towers CG, Thorburn A (2017) Targeting autophagy in cancer. Nat Rev Cancer 17(9):528–542. 10.1038/nrc.2017.53 28751651 10.1038/nrc.2017.53 PMC5975367

[16] Djajadikerta A, Keshri S, Pavel M, Prestil R, Ryan L, Rubinsztein DC (2020) Autophagy induction as a therapeutic strategy for neurodegenerative diseases. J Mol Biol 432(8):2799–2821. 10.1016/j.jmb.2019.12.035 31887286 10.1016/j.jmb.2019.12.035

[17] Kim J, Kundu M, Viollet B, Guan KL (2011) AMPK and mTOR regulate autophagy through direct phosphorylation of Ulk1. Nat Cell Biol 13:132–141. 10.1038/ncb2152 21258367 10.1038/ncb2152 PMC3987946

[18] Lamb CA, Yoshimori T, Tooze SA (2013) The autophagosome: origins unknown, biogenesis complex. Nat Rev Mol Cell Biol 14(12):759–774. 10.1038/nrm3696 24201109 10.1038/nrm3696

[19] Huynh FK, Green MF, Koves TR, Hirschey MD (2014) Measurement of fatty acid oxidation rates in animal tissues and cell lines. Methods Enzymol 542:391–405. 10.1016/B978-0-12-416618-9.00020-0 24862277 10.1016/B978-0-12-416618-9.00020-0 PMC4154315

[20] Frago LM, Canelles S, Freire-Regatillo A, Argente-Arizón P, Barrios V, Argente J, Garcia-Segura LM, Chowen JA (2017) Estradiol uses different mechanisms in astrocytes from the hippocampus of male and female rats to protect against damage induced by palmitic acid. Front Mol Neurosci 10:330–346. 10.3389/fnmol.2017.00330 29114202 10.3389/fnmol.2017.00330 PMC5660686

[21] Granado-Serrano AB, Martín MA, Izquierdo-Pulido M, Goya L, Bravo L, Ramos S (2007) Molecular mechanisms of epicatechin and chlorogenic acid on the regulation of the apoptotic and survival/proliferation pathways in a human hepatoma cell line (HepG2). J Agric Food Chem 55:2020–2027. 10.1021/jf062556x 17286412 10.1021/jf062556x

[22] Palomino OM, Gouveia NM, Ramos S, Martín MA, Goya L (2017) Protective effect of *Silybum marianum* on endothelial cells submitted to high glucose concentration. Planta Med 83:97–103. 10.1055/s-0042-113135 27525510 10.1055/s-0042-113135

[23] Alía M, Ramos S, Mateos R, Bravo L, Goya L (2006) Quercetin protects human hepatoma cell line (HepG2) against oxidative stress induced by tertbutyl hydroperoxide. Toxicol Appl Pharmacol 212:110–118. 10.1016/j.taap.2005.07.014 16126241 10.1016/j.taap.2005.07.014

[24] Klionsky DJ et al (2021) Guidelines for the use and interpretation of assays for monitoring autophagy (4th edition). Autophagy 17(1):1–382 33634751 10.1080/15548627.2020.1797280 PMC7996087

[25] Browne RW, Armstrong D (1998) Reduced glutathione and glutathione disulfide. In Free Radical and Antioxidant Protocols; Armstrong D, Ed.; Methods in Molecular Biology; Humana Press: Totowa, NJ, USA, pp 347–354.

[26] Mauthe M, Orhon I, Rocchi C, Zhou X, Luhr M, Hijlkema KJ, Coppes RP, Engedal N, Mari M, Reggiori F (2018) Chloroquine inhibits autophagic flux by decreasing autophagosome-lysosome fusion. Autophagy 14(8):1435–1455. 10.1080/15548627.2018.1474314 29940786 10.1080/15548627.2018.1474314 PMC6103682

[27] Lu Y, Cheng J, Chen L, Li C, Chen G, Gui L, Shen B, Zhang Q (2015) Endoplasmic reticulum stress involved in high-fat diet and palmitic acid-induced vascular damages and fenofibrate intervention. Biochem Biophys Res Commun 458:1–7. 10.1016/j.bbrc.2014.12.123 25592967 10.1016/j.bbrc.2014.12.123

[28] Dinh QN, Chrissobolis S, Diep H, Chan CT, Ferens D, Drummond GR, Sobey CG (2017) Advanced atherosclerosis is associated with inflammation, vascular dysfunction and oxidative stress, but not hypertension. Pharmacol Res 116:70–76. 10.1016/j.phrs.2016.12.032 28017665 10.1016/j.phrs.2016.12.032

[29] Brown AA, Hu FB (2001) Dietary modulation of endothelial function: implications for cardiovascular disease. Am J Clin Nutr 73:673–686 11273841 10.1093/ajcn/73.4.673

[30] Lu Y, Chen Y, Li R, Liu Q, Wang N, Zhang Y, Li B, Fang Z (2018) Protective effects of Danzhi jiangtang capsule on vascular endothelial damages induced by high-fat diet and palmitic acid. Biomed Pharmacother 107:1631–1640. 10.1016/j.biopha.2018.08.129 30257381 10.1016/j.biopha.2018.08.129

[31] Martín MA, Granado-Serrano AB, Ramos S, Izquierdo-Pulido M, Bravo L, Goya L (2010) Cocoa flavonoids upregulate antioxidant enzymes activity via ERK1/2 pathway to protect against oxidative stress-induced apoptosis in HepG2 cells. J Nutr Biochem 21:196–205. 10.1016/j.jnutbio.2008.10.009 19195869 10.1016/j.jnutbio.2008.10.009

[32] Rodríguez JL, Berrios P, Clavo ZM, Marin-Bravo M, Inostroza-Ruiz L, Ramos-Gonzalez M, Quispe-Solano M, Fernández-Alfonso MS, Palomino O, Goya L (2023) Chemical characterization, antioxidant capacity and anti-oxidative stress potential of South American Fabaceae *Desmodium tortuosum*. Nutrients 15:746. 10.3390/nu15030746 36771451 10.3390/nu15030746 PMC9921092

[33] Thellung S, Corsaro A, Nizzari M, Barbieri F, Florio T (2019) Autophagy Activator drugs: a new opportunity in neuroprotection from misfolded protein toxicity. Int J Mol Sci 20:901. 10.3390/ijms20040901 30791416 10.3390/ijms20040901 PMC6412775

[34] Gouveia NM, Ramos S, Martín MA, Spindola F, Goya L, Palomino OM (2017) *Vochysia rufa* stem bark extract protects endothelial cells against high glucose damage. Medicines 4(9):4010009. 10.3390/medicines4010009 10.3390/medicines4010009 PMC5597076 28930225

[35] Ferreira-Martins T, Palomino O, Álvarez-Cilleros D, Ramos S, Goya L (2020) Cocoa flavanols protect human endothelial cells from oxidative stress. Plant Foods Hum Nutr 75:161–168. 10.1007/s11130-020-00807-1 32185628 10.1007/s11130-020-00807-1

[36] Richieri GV, Kleinfeld AM (1995) Unbound free fatty acid levels in human serum. J Lipid Res 36:229–240 7751810

