# Supplementary material for: Does autophagy play a key role in the protective effect of oleic acid against oxidative stress in endothelial cells?
Source: Mol Cell Biochem. 2025 Oct 15;481(1):387–99. doi: 10.1007/s11010-025-05410-z (PMC12906551; doi:10.1007/s11010-025-05410-z)
Supplement: Supplementary file 1 — Supplementary file1 (PPTX 5075 kb) [file 11010_2025_5410_MOESM1_ESM.pptx]

## Slide 1
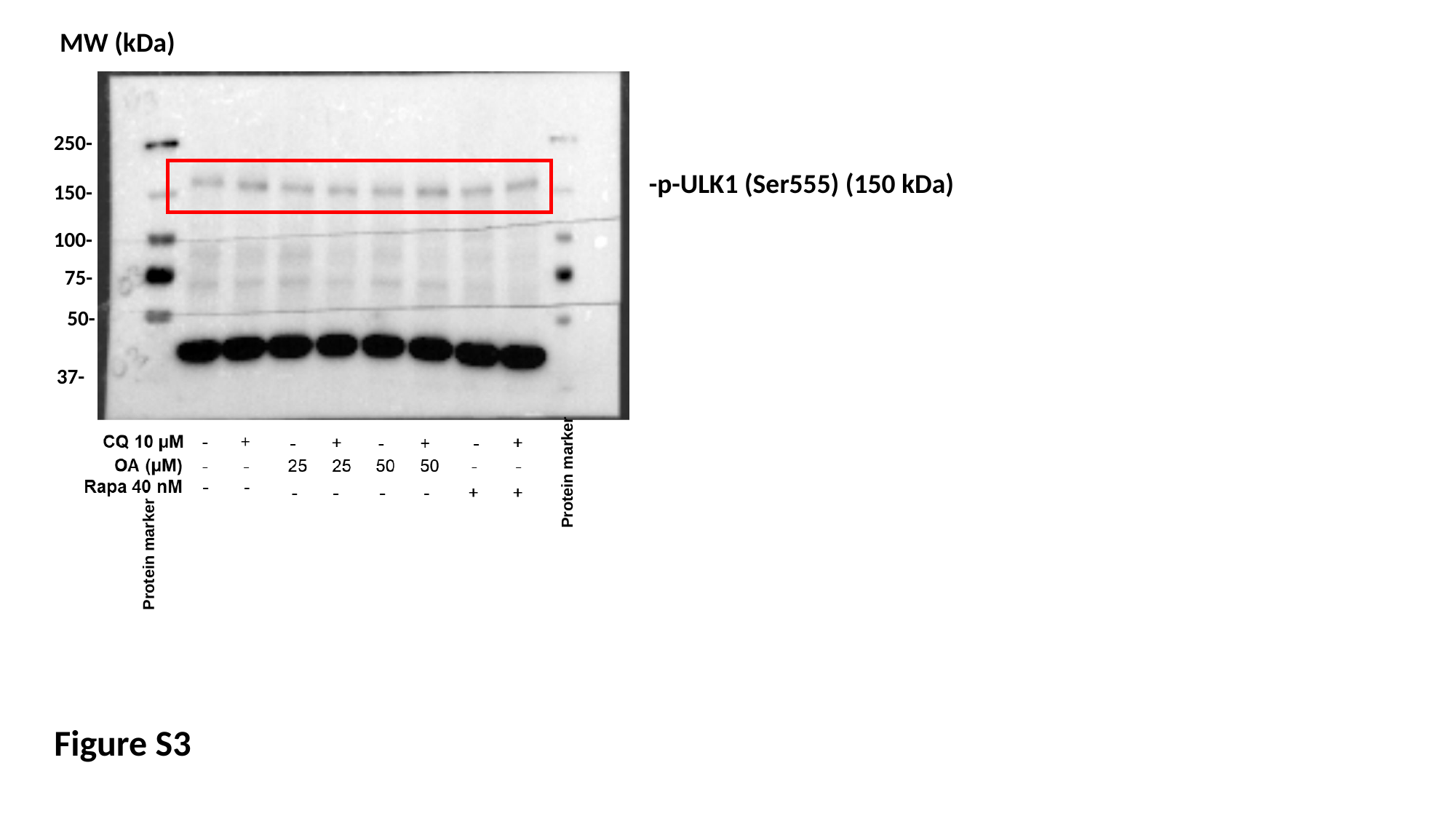

MW (kDa)
250-
-p-ULK1 (Ser555) (150 kDa)
150-
100-
75-
50-
37-
Protein marker
Protein marker
Figure S3

## Slide 2
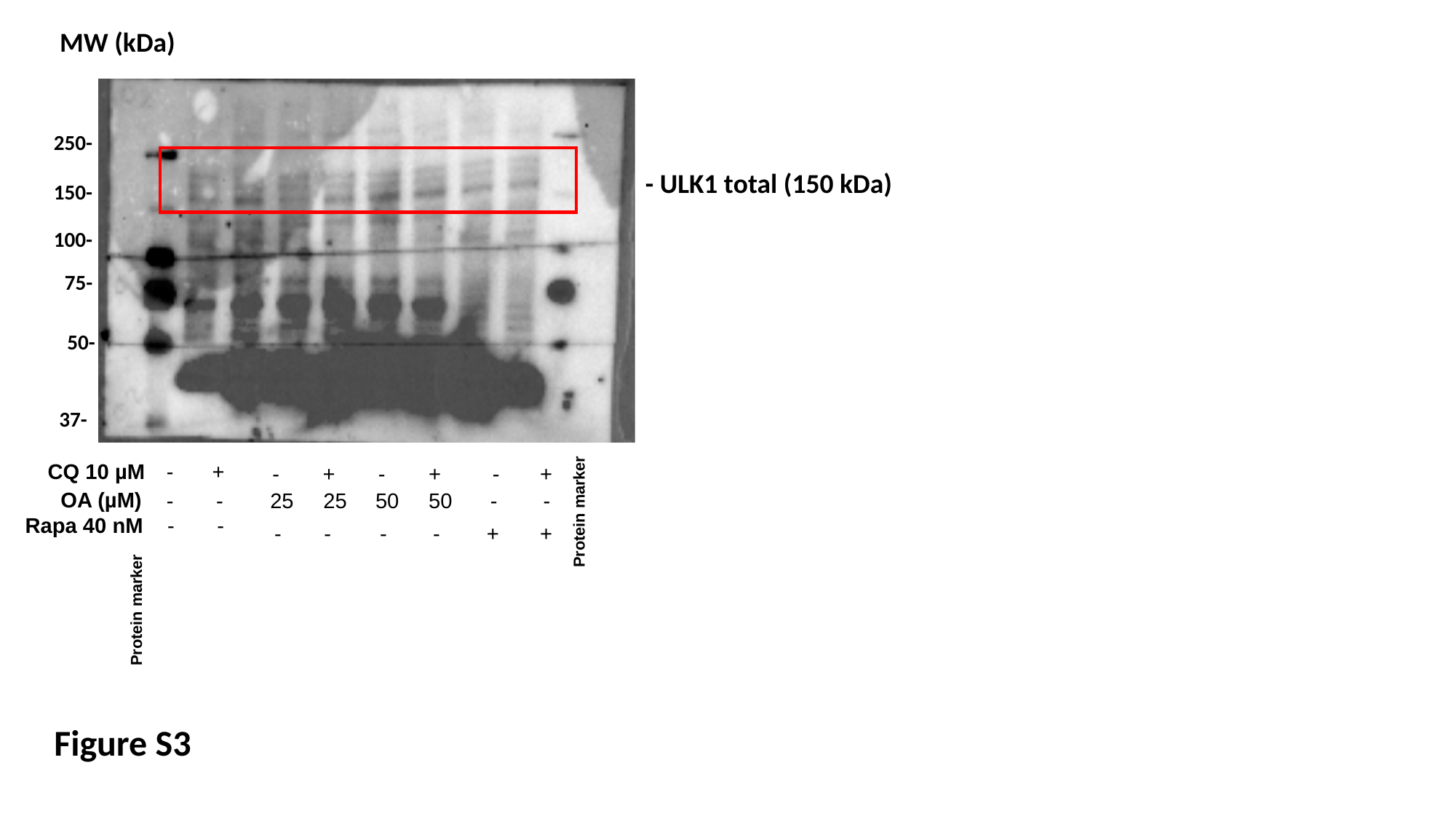

MW (kDa)
250-
- ULK1 total (150 kDa)
150-
100-
75-
50-
37-
CQ 10 µM
-
+
-
+
-
-
+
+
OA (µM)
-
-
25
25
50
50
-
-
Rapa 40 nM
-
-
-
-
-
-
+
+
Protein marker
Protein marker
Figure S3

## Slide 3
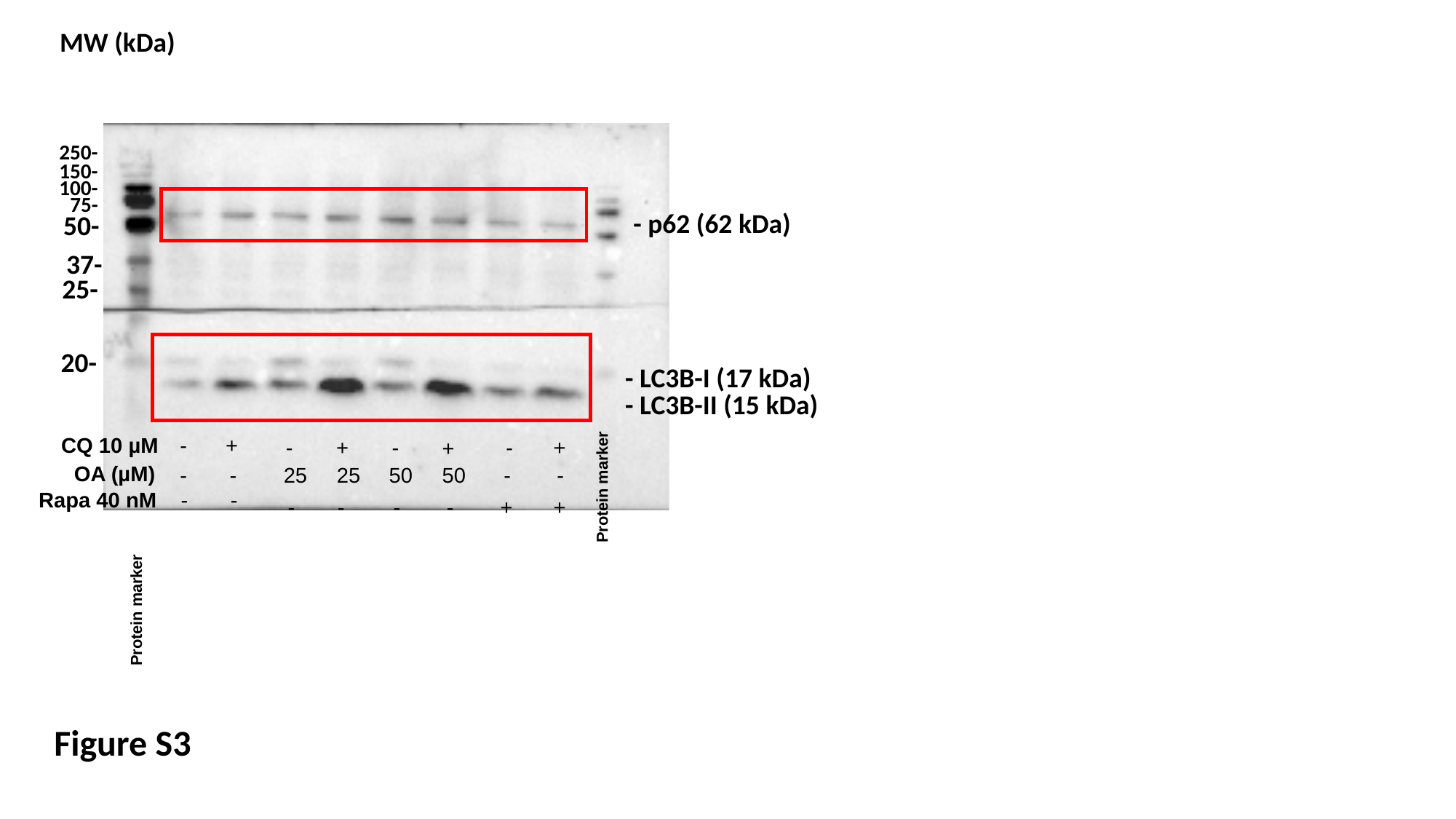

MW (kDa)
250-
150-
100-
75-
- p62 (62 kDa)
50-
37-
25-
20-
- LC3B-I (17 kDa)
- LC3B-II (15 kDa)
CQ 10 µM
-
+
-
+
-
-
+
+
OA (µM)
-
-
25
25
50
50
-
-
Rapa 40 nM
-
-
-
-
-
-
+
+
Protein marker
Protein marker
Figure S3

## Slide 4
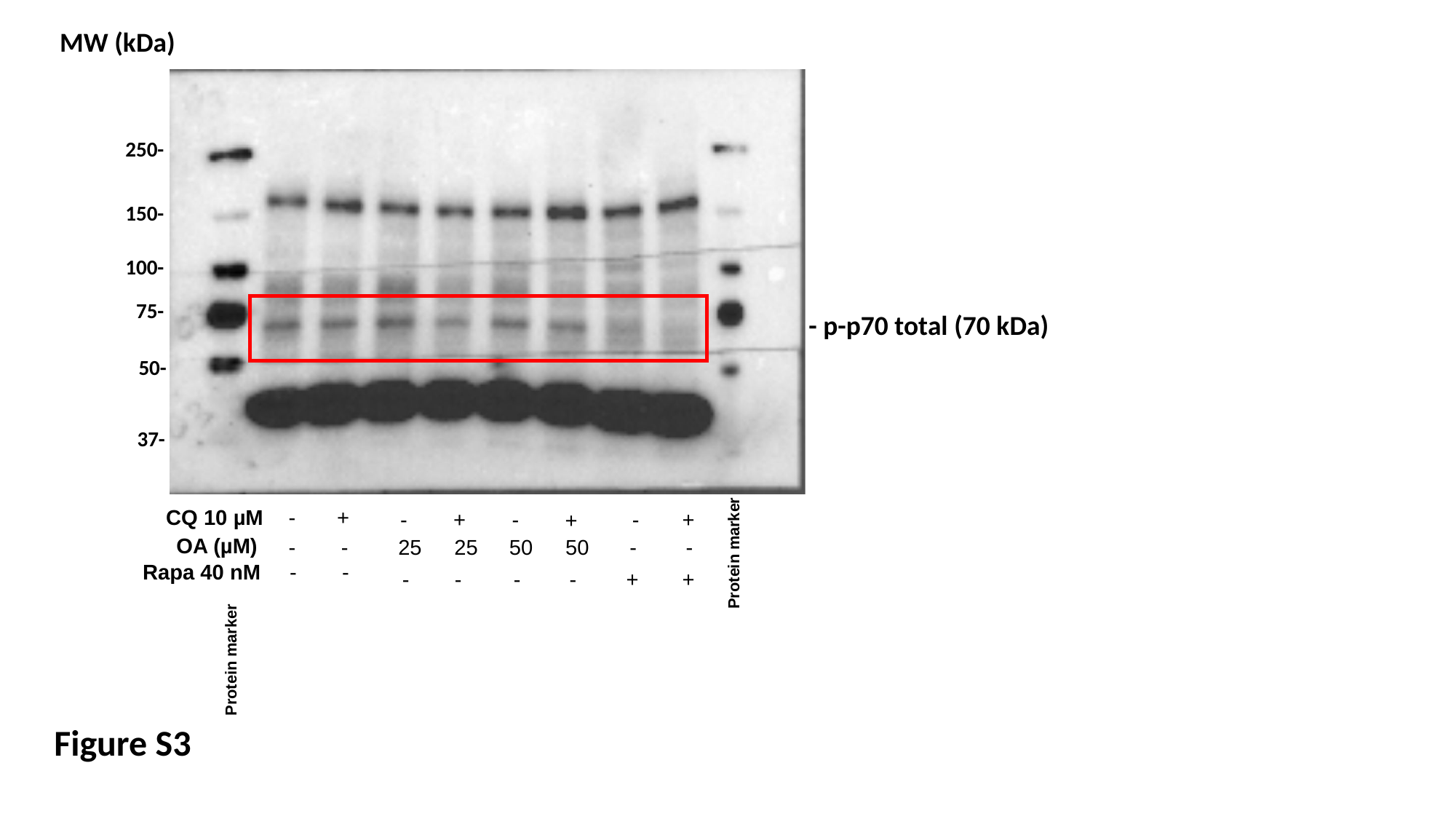

MW (kDa)
250-
150-
100-
75-
- p-p70 total (70 kDa)
50-
37-
CQ 10 µM
-
+
-
+
-
-
+
+
OA (µM)
-
-
25
25
50
50
-
-
Rapa 40 nM
-
-
-
-
-
-
+
+
Protein marker
Protein marker
Figure S3

## Slide 5
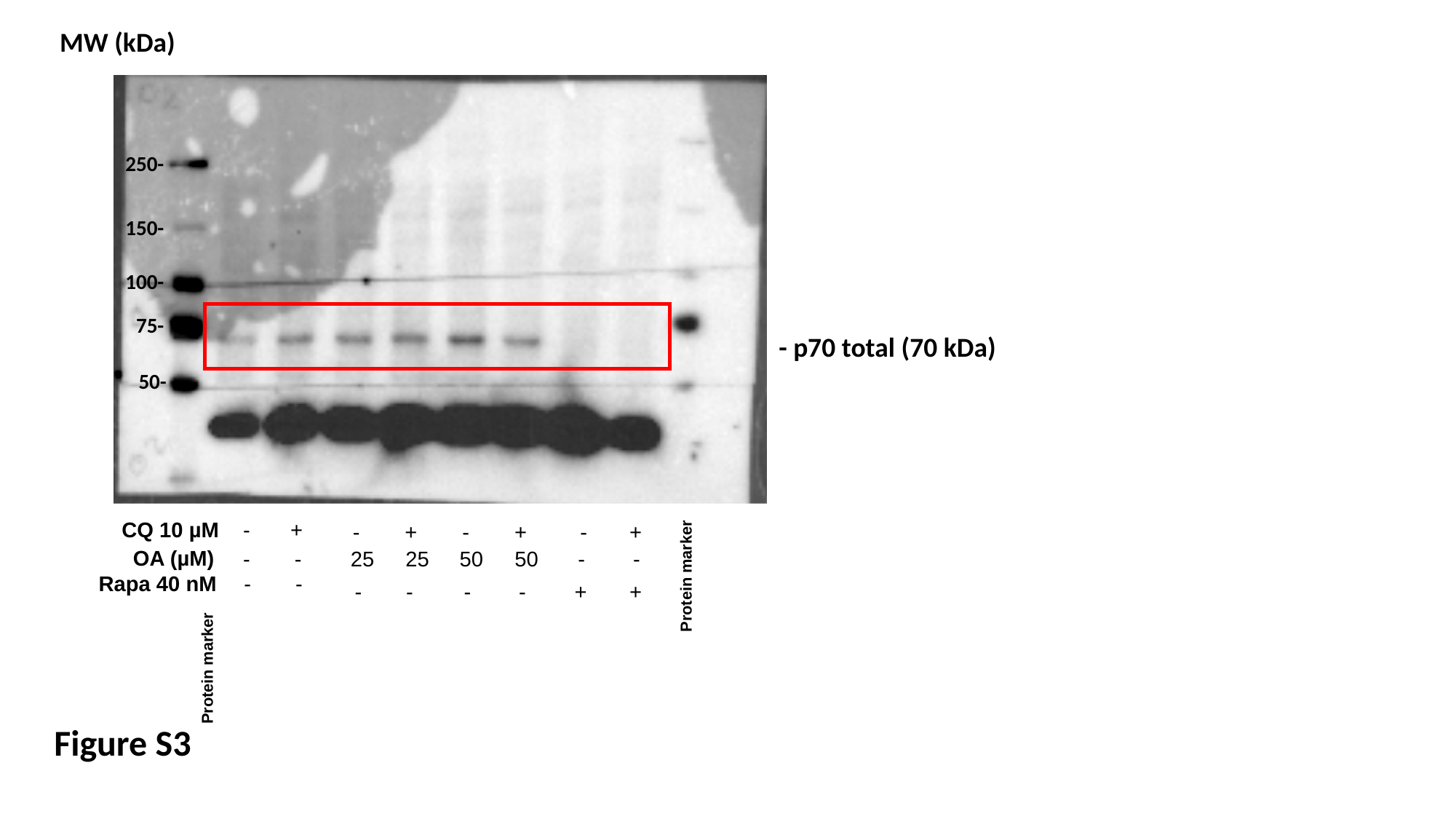

MW (kDa)
250-
150-
100-
75-
- p70 total (70 kDa)
50-
CQ 10 µM
-
+
-
+
-
-
+
+
OA (µM)
-
-
25
25
50
50
-
-
Rapa 40 nM
-
-
-
-
-
-
+
+
Protein marker
Protein marker
Figure S3

## Slide 6
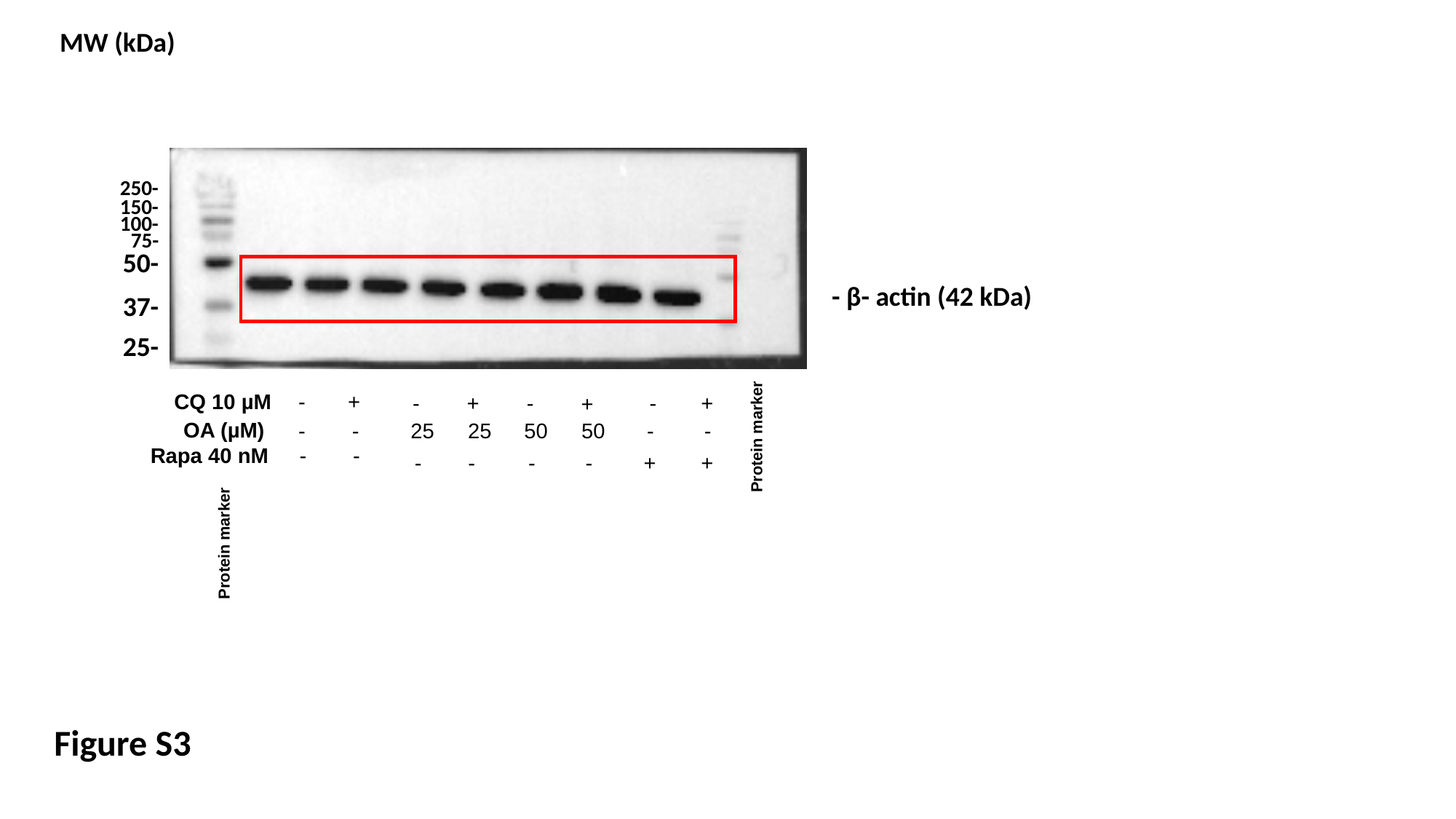

MW (kDa)
250-
150-
100-
75-
50-
- β- actin (42 kDa)
37-
25-
CQ 10 µM
-
+
-
+
-
-
+
+
OA (µM)
-
-
25
25
50
50
-
-
Rapa 40 nM
-
-
-
-
-
-
+
+
Protein marker
Protein marker
Figure S3

## Slide 7
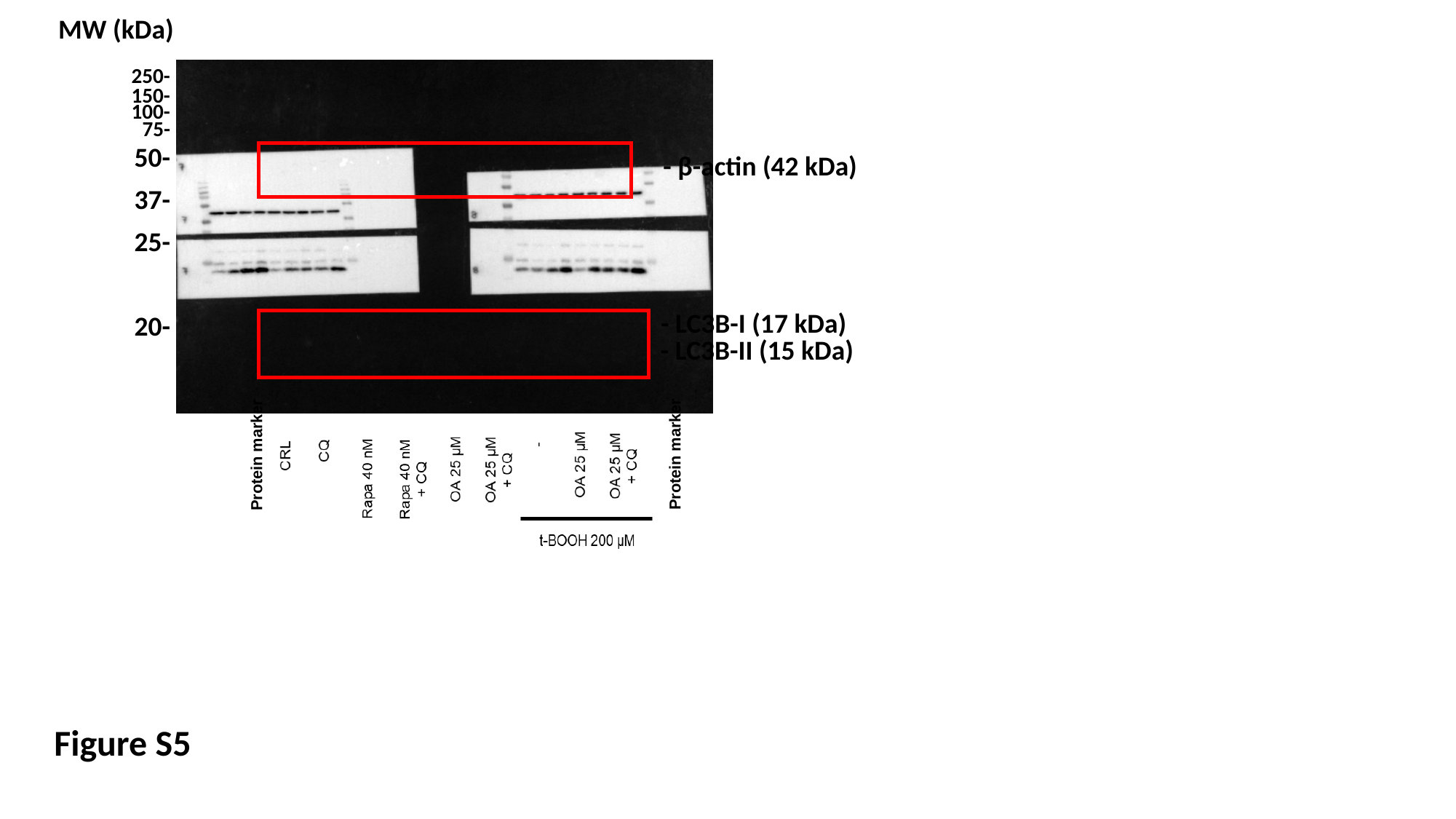

MW (kDa)
250-
150-
100-
75-
50-
- β-actin (42 kDa)
37-
25-
- LC3B-I (17 kDa)
20-
- LC3B-II (15 kDa)
Protein marker
Protein marker
Figure S5
